# Investigating a Genetic Link Between Alzheimer’s Disease and CADASIL-Related Cerebral Small Vessel Disease

**DOI:** 10.1007/s12035-022-03039-3

**Published:** 2022-09-29

**Authors:** Paul J. Dunn, Rodney A. Lea, Neven Maksemous, Robert A. Smith, Heidi G. Sutherland, Larisa M. Haupt, Lyn R. Griffiths

**Affiliations:** 1 Centre for Genomics and Personalised Health, Genomics Research Centre, School of Biomedical Sciences, Faculty of Health, Queensland University of Technology (QUT), 60 Musk Ave, Kelvin Grove, QLD 4059 Australia; 2 Faculty of Health Sciences and Medicine, Bond University, 14 University Drive, Robina, QLD 4226 Australia

**Keywords:** Whole exome sequencing, Cerebral small vessel disease, CADASIL, Alzheimer’s disease, Burden test

## Abstract

Monogenic forms of Alzheimer’s disease (AD) have been identified through mutations in genes such as *APP, PSEN1,* and *PSEN2*, whilst other genetic markers such as the *APOE* ε carrier allele status have been shown to increase the likelihood of having the disease. Mutations in these genes are not limited to AD, as *APP* mutations can also cause an amyloid form of cerebral small vessel disease (CSVD) known as cerebral amyloid angiopathy, whilst *PSEN1* and *PSEN2* are involved in *NOTCH3* signalling, a process known to be dysregulated in the monogenic CSVD, cerebral autosomal dominant arteriopathy with subcortical infarcts and leukoencephalopathy (CADASIL). The overlap between AD genes and causes of CSVD led to the hypothesis that mutations in other genes within the PANTHER AD–presenilin pathway may be novel causes of CSVD in a cohort of clinically suspicious CADASIL patients without a pathogenic *NOTCH3* mutation. To investigate this, whole exome sequencing was performed on 50 suspected CADASIL patients with no *NOTCH3* mutations, and a targeted gene analysis was completed on the PANTHER. *ERN1* was identified as a novel candidate CSVD gene following predicted pathogenic gene mutation analysis. Rare variant burden testing failed to identify an association with any gene; however, it did show a nominally significant link with *ERN1* and *TRPC3.* This study provides evidence to support a genetic overlap between CSVD and Alzheimer’s disease.

## Background

Cerebral autosomal dominant arteriopathy with subcortical infarcts and leukoencephalopathy (CADASIL) is the most common monogenic form of cerebral small vessel disease (CSVD) [1]. It has been originally estimated to have a prevalence of 2–4 per 100,000; however, later studies of population data from the Genome Aggregation Database (gnomAD) estimate that it may be up to 1 in 300 [2, 3]. CADASIL predominantly affects the central nervous system (CNS) and symptoms include recurrent ischaemic events, migraine, mood disturbances, progressive cognitive decline/vascular dementia, and sometimes seizures [4, 5].

Mutations in *NOTCH3* which alter cysteines within exons 2–24 encompassing the epidermal growth factor-like repeats (EGFRs) were originally identified as causal in CADASIL patients [6]. The theorised pathogenic role is that these mutations result in an odd number of cysteine residues in one of the EGFRs, resulting in disrupted disulphide bond formation which impacts NOTCH3 signalling. Studies have shown that NOTCH3 signalling is important in brain and retinal vasculature, where *Notch3* deficient adult mice showed loss of vessel integrity resulting from vascular smooth muscle cell (VSMC) degradation and apoptosis [7]. Furthermore, these Notch3 deficient mice showed an increased likelihood of haemorrhages and a loss of blood–brain barrier function [7, 8]. Notch signalling is highly conserved across species and involves interactions with ligands (JAG1, DLL1, DLL2) binding to the extracellular domain of the NOTCH3 protein. The protein then undergoes a cleavage event by ADAM10 at the S2 cleavage site, and then by the ɣ-secretase complex made up of Presenilin-1 (PSEN1), Presenilin-2 (PSEN2), Nicastrin (NCSTN), anterior pharynx defective 1 (APH-1), and presenilin enhancer 2 (PEN2) [9]. This second cleavage event results in the release of the NOTCH3 intracellular domain (N3ICD) into the cytoplasm where it is trafficked into the nucleus and can act as a transcriptional regulator. NOTCH3 signalling is primarily limited to the VSMCs and as such mutations in *NOTCH3* cause a gradual degeneration of these cells leading to recurrent infarcts and gradual development of vascular dementia [10].

Interestingly, the ɣ-secretase complex that is involved in the NOTCH cleavage steps and is also known to play a role in the cleavage of apolipoprotein precursor protein (APP). Along with mutations in *PSEN1* and *PSEN2, APP* is one of the most studied genes known to cause Alzheimer’s disease. Mutations in *APP* have also been associated with cerebral amyloid angiopathy (CAA), a condition that results in amyloid deposits accumulating around VSMCs in the abluminal aspect of the intimal layers of the blood vessels [11, 12]. In a similar mechanism to CADASIL, this condition can cause cerebral haemorrhages, brain bleeds, and dementia [13–15]. Due to the phenotypic overlap of these conditions and that the pathways involved are linked via the ɣ-secretase complex, we hypothesised that there are potential causative mutations in genes within the AD-presenilin pathway that may be causing a CADASIL-like phenotype, or a novel form of CADASIL-related CSVD. Therefore, we investigated whether rare and functional variants in Alzheimer-related genes from the protein analysis through evolutionary relationships (PANTHER) AD-Presenilin pathway were present in patients with suspected CADASIL, but without *NOTCH3* mutations.

## Methods

### Patient Cohort

The study cohort comprised patients who were initially referred by neurologists to the Genomics Research Centre (GRC) diagnostic testing facility for CADASIL testing (Targeted *NOTCH3* next-generation sequencing). From these, 50 samples were selected based on the previous testing using the GRC custom 5-gene panel (*CACNA1A, ATP1A2, SCN1A, NOTCH3, KCNK18*) where no causative mutation was identified in *NOTCH3* or in any of the other genes on the panel [16]. Ethical approval through the Queensland University of Technology (QUT) human research ethic council (HREC), and appropriate consents for the patient cohort, are already in place (approval number 1800000611).

### DNA Extraction and Whole Exome Sequencing

Genomic DNA was extracted from peripheral blood lymphocytes using the QIAGEN QIAcube™ (Venlo, Netherlands). Aliquots of DNA were quantified and checked for quality using a Thermo Fisher Scientific Nanodrop Spectrophotometer 8000 (Waltham, MA, USA) and diluted to a concentration of ~ 20 ng/µL for whole exome sequencing using the Ion AmpliSeq™ Exome RDY-kits (Carlsbad, CA., USA) for library preparation, according to manufactures’ instructions (MAN0010084). Completed libraries were quantified using QIAGEN Qubit™ v.3 (Venlo, Netherlands) and combined at an equimolar concentration of 100 pM. Template preparation, enrichment, and chip loading were performed using the Ion P1™ Hi-Q™ Chef Kit (Cat. Number A27198) and 540 Chips (Cat. Number A30011) on the Applied Biosystems Ion Chef (Carlsbad, CA, USA) targeted at 200 bp lengths. Sequencing was performed using the Ion Proton™ and Ion S5 + platforms with sequencing alignment (Hg19) and variant calling was completed via the Ion Torrent™ software (Carlsbad, CA, USA).

### Targeted Candidate Mutation Analysis and Curation

Analysis was completed through merging 50 variant call format (vcf) files using the bcf-tools vcf-merge function and completing variant annotation using ensemble-vep [17]. The merged vcf file was then filtered based on a list of 131 genes that are part of the PANTHER Alzheimer disease – presenilin pathway (Table 1) from (http://www.pantherdb.org/list/list.do?numPerPage=200&save=yes&searchModType=numperpage&listType=1). These genes were identified through selecting the components part of the Alzheimer’s disease – presenilin pathway (Accession: P00004) and converting the list to genes and filtering for *homo* *sapiens*. Candidate variants were filtered based on pathogenic in silico prediction tools such as MutationTaster, SIFT, PolyPhen, and PredictSNP2 (which includes CADD, DANN, FATHMM, FunSeq2, and GWAVA scores) as well as an overall gnomAD MAF < 0.001 [18–22]. Variants were excluded if there were two or more in silico tools that identified the variant as benign/tolerated. Variants were further checked in disease and variant databases such as ClinVar, dbSNP, and for previous pathogenicity and disease-causing curations. Finally, variants were checked for the candidacy of causing disease based on gene ontology, genetic interactions, and gene expression.

**Table 1:** PANTHER Alzheimer’s disease – presenilin pathway genes used for analysis listed in alphabetical order

ACTA1	CDH1	FZD6	MMP12	NECTIN1	TCF7	WNT4
ACTA2	CDH3	FZD7	MMP13	NOTCH1	TCF7L1	WNT5A
ACTB	CTNNA1	FZD8	MMP14	NOTCH2	TCF7L2	WNT5B
ACTBL2	CTNNA2	FZD9	MMP15	NOTCH2NLB	TRIM2	WNT6
ACTBL3	CTNNB1	GSK3B	MMP16	NOTCH3	TRIM3	WNT7A
ACTC1	DVL1	HVEC	MMP17	NOTCH4	TRPC1	WNT7B
ACTG1	DVL1P1	JUP	MMP19	PCSK1	TRPC3	WNT8A
ACTG2	DVL2	KAT5	MMP2	PCSK2	TRPC4	WNT8B
ACTR2	DVL3	KAT7	MMP20	PCSK4	TRPC5	WNT9A
ADAM17	ERBB4	LEF1	MMP21	PCSK5	TRPC6	WNT9B
AFDN	ERN1	LRP1	MMP23B	PCSK6	TRPC7	
APBB1	ERN2	LRP1B	MMP24	PCSK7	WNT1	
APBB2	FSTL1	LRP2	MMP25	POTEK	WNT10A	
APBB3	FURIN	LRP3	MMP27	POTEKP	WNT10B	
APH1A	FZD1	LRP4	MMP28	PSEN1	WNT11	
APH1B	FZD10	LRP5	MMP7	PSEN2	WNT16	
APP	FZD2	LRP5L	MMP8	PSENEN	WNT2	
BACE1	FZD3	LRP6	MMP9	PVRL1	WNT2B	
BACE2	FZD4	MLLT4	N2N	RBPJ	WNT3	
CD44	FZD5	MMP1	NCSTN	RBPJL	WNT3A	

An additional analysis of *APOE* ε carrier allele status was also completed. This involved extracting the rs429358 and rs7412 variants identified in *APOE* from WES data for each individual [23]. The rate of each genotype identified was then used to determine if there is an increased risk for Alzheimer’s disease in this cohort and was compared to gnomAD population data.

### Burden Testing the Alzheimer’s Disease Gene Pathway

**Fig. 1:**
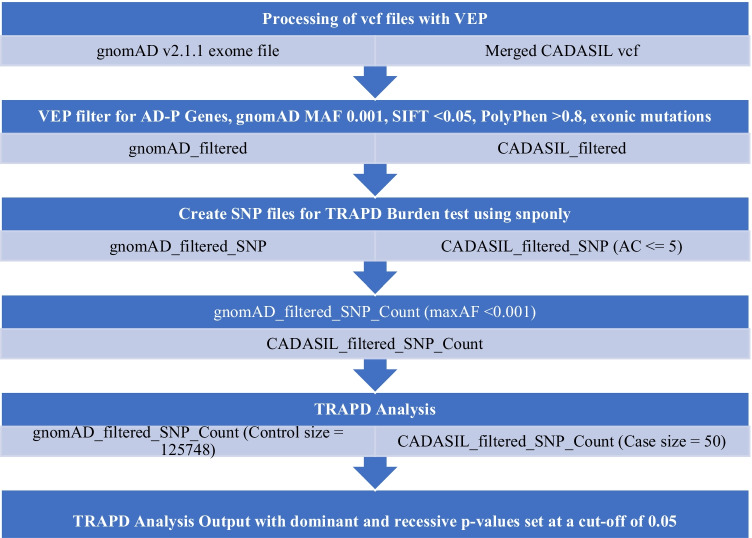
Analysis pipeline for using the TRAPD burden test protocol with added pre-processing steps. Values in brackets are where there are minor differences completed for each file

Following candidate mutation identification, a rare-variant association test using the TRAPD software was also completed to determine if there was any increase in the number of rare variants in the Alzheimer’s disease—presenilin pathway genes [24]. This test involved using a modified analysis pipeline (Fig. 1) of the merged and annotated vcf file with just the AD-presenilin genes and counting the number of rare variants (gnomAD global MAF < 0.001).

Variants were also filtered in if there was a score of ≤ 0.05 for SIFT and a score of ≥ 0.8 for PolyPhen had a coverage depth of ≥ 20X. To further remove the likelihood of artefactual results, there was also a threshold set for an overall allele count (AC) < 5 for each variant in the merged vcf file. To ensure the same regions of the genes were covered, both the gnomAD and CADASIL-related CSVD vcf files were filtered to only include non-intronic variants. The TRAPD burden test scripts initially involved creating a variant (SNP) file for both the gnomAD v2.1.1 filtered vcf and the CADASIL merged filtered vcf file. The number of alleles for each SNP identified was then counted and collated into its associated gene count, where a second filter for the gnomAD controls was added to make sure that only variants seen in less than 1 in 1000 gnomAD cases were counted. This was done in accordance with the TRAPD burden test guidelines to further remove bias from the system. Finally, a burden test was run using Fisher’s exact test to identify a greater burden in variants identified across genes in the CADASIL case-cohort versus the control cohort. There were two *p* values calculated for each gene based on either a predicted Mendelian autosomal dominant or autosomal recessive inheritance pattern.

## Results

WES was conducted for 50 patients referred for CADASIL testing, but negative for mutations in *NOTCH3*. Overall, the initial analysis of the WES data identified *n* = 20 mutations across *n* = 15 PANTHER Alzheimer’s disease – presenilin pathway genes (Table 2). This included a rare mutation in *APP* in DGR349, where mutations in this gene are known to cause Alzheimer’s disease and/or cerebral amyloid angiopathy. There were *n* = 2 mutations in *SMPD1* across two separate samples (DGR330, DGR332) which encodes for Sphingomyelin phosphodiesterase 1. There were also three samples with mutations in *ERN1* (DGR024, DGR037, and DGR323) which encodes for Endoplasmic Reticulum to Nucleus Signalling and *TRPC4* (DGR321, DGR343, DGR366) which encodes for Transient Receptor Potential Cation Channel Subfamily C Member 4. Mutations in *NOTCH1* and *NOTCH2* were also identified in this analysis showing potential variants of interest in other NOTCH family genes.

**Table 2:**
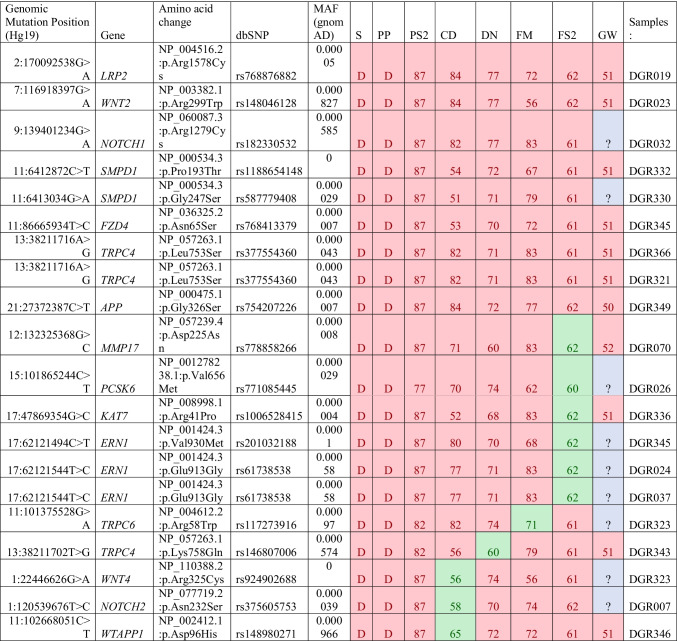
Mutations identified through targeted WES analysis focussing on the PANTHER Alzheimer’s disease

Presenilin pathway genes are stratified according to the number of tools showing a damaging/deleterious effect (red), an unknown effect (blue) and a benign/tolerated effect (green). In silico pathogenicity prediction tools such as SIFT (S), PolyPhen (PP), PredictSNP2(PS2), CADD (CD), DANN (DN), FATHMM (FM), FunSeq2 (FS2), and GWAVA (GW) were used to stratify mutations. Only gene mutations with a MAF < 0.001 were used for this table

The ApoE homozygous rs429358 CC variants were seen at 10 times the rate of the gnomAD population control (Table 3) and heterozygous rs7412 CT variants occurred 1.66 times more frequently in the case-cohort compared to the general gnomAD population observing this genotype [24]. There was a total of 10% (*n* = 5/50) CADASIL referred individuals that have the *APOE*-ε4/ε4 genotype (Table 4). The remaining genotypes identified that the ε3/ε4 and ε3/ε3 were observed in 42% (*n* = 21/50) and 38% (*n* = 19/50) respectively of the CADASIL-CSVD population, while the ε2/ε3 and ε2/ε4 genotypes were seen in 8% (*n* = 4/50) and 2% (*n* = 1/50) respectively (Table 4). Interestingly, this showed that 50% of the CADASIL-related CSVD cohort had at least one *APOE4* AD risk allele.

**Table 3:** Investigation of the rate of APOE variants in the CADASIL-related CSVD cohort compared to the rate observed in gnomAD

		CADASIL cohort	CADASIL rate	gnomAD cohort	gnomAD rate
rs429358	TT	25	0.5	172283	0.857
	TC	20	0.4	26546	0.132
	CC	5	0.1	2091	0.010
	Total	50	1	200920	
rs7412	CC	45	0.9	157082	0.935
	CT	5	0.1	10531	0.063
	TT	0	0	465	0.003
	Total	50	1	168078

**Table 4:** Distribution of APOE genotypes across the CADASIL-CSVD cohort

APOE genotype	rs429358 allele	rs7412 allele	Number	Percentage
ε2/ε2	TT	TT	0	0%
ε2/ε3	TT	CT	4	8%
ε2/ε4	CT	CT	1	2%
ε3/ε3	TT	CC	21	42%
ε3/ε4	CT	CC	19	38%
ε4/ε4	CC	CC	5	10%
		Total:	50	100%

Rare variant association testing using the TRAPD software failed to identify an increased pathogenic rare variant burden in the AD-Presenilin genes within the CADASIL-like CSVD cohort compared to the GnomAD population that passed multiple testing. However, two genes (*ERN1* *p* = 0.0015 FDR = 0.38; *TRPC3* *p* = 0.015 FDR = 1) were found to be nominally significant using the autosomal dominant model (Table 5). There was no nominal association identified in the recessive model.

**Table 5:** Statistically significant genes identified from the TRAPD burden test

Gene	Case HET	Case CH	Case HOM	Case total AC	Control HET	Control HOM	Control total AC	*p* DOM	FDR DOM
*ERN1*	5	0	0	5	2112	0	2112	**0.0015**	0.3735
*TRPC3*	3	0	0	3	1298	0	1298	**0.0150**	1

Abbreviations: *HET* heterozygous; *CH* compound heterozygous, *HOM* homozygous; *AC* allele count; *DOM* dominant inheritance pattern; *REC* recessive inheritance pattern; *FDR* false discovery rate

## Discussion

**Fig. 2:**
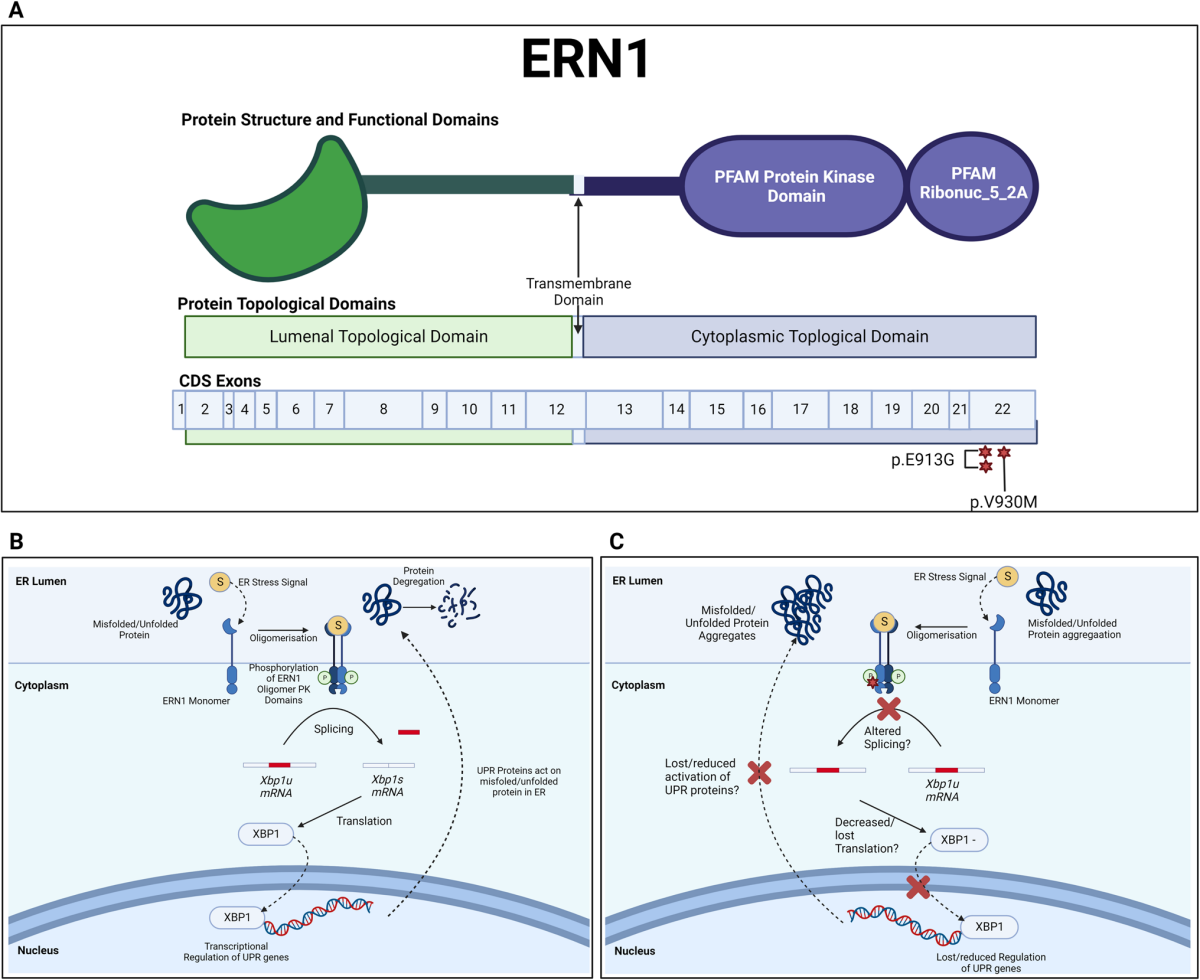
ERN1 protein structure and potential impact of identified variants. Panel of ERN1 protein structure and role in the unfolded protein response (UPR) cycle. Panel A. shows the ERN1 protein with its topological domains (Lumenal, Transmembrane, and Cytoplasmic), functional domains (Protein Kinase and Ribonuc_5_2A), and the CDS exonic locations that encode for each domain. The two variants identified in this study show the Ribonuc_5_2A functional domain may be affected. Panel B. shows the role of ERN1 in the UPR cycle and is adapted from Li et. al. (2019) [28]. The Ribonuc_5_2A domain at the C-terminal of the protein is involved in splicing XBP1 mRNA allowing for the protein to be translated to act as a transcriptional regulator of UPR genes. Panel C. predicts the potential downstream functional effects of the ERN1 gene variants in splicing XBP1 mRNA and altering the transcriptional regulation of UPR genes

Candidate mutation analysis based on MAF thresholds and in silico prediction tools of the AD-presenilin genes identified 20 mutations across 15 genes which were further investigated as novel candidates for CSVD. *ERN1* may be the strongest novel candidate for CSVD pathology as there were five variants identified across the CADASIL-CSVD cohort that were considered significant, that included the three candidate mutations (Table 2). The *ERN1* association identified from the TRAPD burden test also mimics an exome-wide association study of *APOE* ε4 non-carrier Alzheimer’s disease which found a rare synonymous variant in *ERN1* (rs56201815) as being associated with late-onset Alzheimer’s disease (LOAD) [25]. *ERN1* encodes for a resident transmembrane endoplasmic reticulum (ER) protein which has both kinase and endonuclease domains (Fig. 2) that works as a key sensor for the ER unfolded protein response [26, 27]. This response activates the genes involved in the degradation of misfolded proteins, regulating protein synthesis and activating molecular chaperones [27].

Misfolded proteins that aggregate are a common feature of CSVDs such as CADASIL (*NOTCH3* mutations), Collagen, type IV, alpha-related small vessel disease (*COL4A1* and *COL4A2* mutations), and CAA (*APP* – mutations) [29–32]. As such, *ERN1* dysfunction may result in increased formation and accumulation of misfolded proteins, and aggregation in the intra- or extracellular spaces in the vessel walls contributing to the pathology of CADASIL and related CSVD pathologies. Interestingly, a previous study found that inhibited expression of *ERN1* reduced the levels of APP in cortical and hippocampal areas in AD mice, restoring their learning and memory capacity [33]. Whilst the mutations identified have not been confirmed to affect function, these studies indicate that dysfunctional ERN1 may have a contributory or causative role in CSVD pathology.

In humans, pharmacological modulation of the ERN1 protein can counteract metaflammation (ubiquitous low levels of inflammation throughout the body) a process that is associated with blood vessel–affecting diseases such as atherosclerosis, chronic heart disease, and diabetes mellitus [34]. ERN1 signalling has also been identified to increase cell cytotoxicity and apoptosis in neuronal ischaemic injury [35]. However, some studies have also identified that ERN1 signalling also exerts a neuroprotective response which supports that the ERN1-pathway may be pro-survival in ischaemic stroke through the activation of chaperone proteins [36]. While this gene did not pass multiple testing using TRAPD burden software, this analysis was based on a low sample size and *ERN1* remains an interesting candidate to investigate further in CSVD and neurodegenerative phenotypes [37, 38].

The amyloid cascade hypothesis for AD is based on the accumulation of amyloid-β peptide as the key driver of early-onset AD [39]. This hypothesis is based on disease-causing mutations identified in *PSEN1, PSEN2,* and *APP,* as well as the ApoE variants found to increase the risk of developing early onset AD. The role of PSEN1 and PSEN2 in the ɣ-secretase complex, as well as *APP* being a known cause of the CAA made these genes interesting targets to investigate further in this study. Furthermore, the link between CSVD and AD is constantly expanding, with some studies showing that CSVD can occur with AD, indicating that the two diseases are interconnected [40, 41]. Within this suspected CADASIL cohort, it was observed that five participants (10%) had the *APOE*-ε4/ε4 genotype and 25 participants (50%) had at least one *APOE-ε4* allele. Interestingly, the percentage of *APOE-ε4* carriers in the CADASIL-related CSVD cohort is in line with what is expected to see in global AD patient cohorts which can range from 38.9–64.4% [42, 43]. This may represent a cause of some of the CADASIL-related symptoms, as *APOE-ε4* carriers show a significant decline in cognitive function by age 69, with the strongest association being seen in *APOE*-ε4/ε4 individuals [44]. This may mean that some suspected CADASIL patients are presenting with Alzheimer’s disease and may have comorbidities that mimic a CADASIL phenotype. It would be interesting to investigate this further in other cohorts.

The heterozygous *APP* p.Gly326Ser mutation may be causative of the amyloid-type CSVD, CAA—an autosomal dominant condition where amyloid progressively deposits in the cerebral blood vessel walls causing degenerative vascular changes and spontaneous cerebral haemorrhages, ischaemic lesions, and dementia [12]. Furthermore, mutations in *APP* are also well documented to cause AD [45, 46]. The mutation was predicted as disease-causing across all forms of in silico pathogenicity prediction tools (SIFT, PolyPhen2, MutationTaster, PredictSNP2, CADD, DANN, FATHMM, and FunSeq2) and has a MAF of 2.4e^−5^. The computational evidence together with the MAF may be indicative of CAA/AD. The presence of the white matter hyperintensities that were “characteristic of CADASIL” make CAA the most-plausible cause of CSVD in DGR349. Interestingly, this individual also had the heterozygous *APOE*-*ε3*/*ε4* genotype. Together, this may have increased the likelihood of AD or CAA in this individual.

Interestingly, three *TRPC* gene paralogs (*TRPC3, TRPC4,* and *TRPC6*) were identified within this study. *TRPC3* was identified as nominally significant through the TRAPD burden test although didn’t pass multiple testing. Interestingly, this was due to three individuals which had the same variant identified (4:122872901A > G) in an untranslated region of the gene, so the functional consequence of these is not clear. In contrast, *TRPC4* and *TRPC6* were identified with candidate predicted pathogenic variants (*TRPC4* rs377554360 -NP_057263.1:p.Lys758Gln and rs146807006—NP_057263.1:p.Leu753Ser; *TRPC6* rs117273916—NP_004612.2:p.Arg58Trp). Further investigation of these mutations via MutationTaster classified the *TRPC6* rs117273916 as polymorphism and therefore as likely harmless, despite it recognising that splice sites may be affected, whilst the *TRPC4* mutations were classified as disease-causing by MutationTaster software. The two *TRPC4* mutations (NP_057263.1:p.Lys758Gln and NP_057263.1:p.Leu753Ser) were seen in three separate individuals where NP_057263.1:p.Leu753Ser was identified as pathogenic across all in silico prediction tools and NP_057263.1:p.Lys758Gln was identified as pathogenic across all tools, apart from DANN. Both mutations in *TRPC4* are within the topological domain of the protein in the region that binds to ITPR1, ITPR2, and ITPR3. A role for *TRPC4* has not been implicated CSVD; however, it has been found to play a role in regulating blood–brain barrier function and is hypothesised to be crucial for oedema formation in ischaemic stroke [47–49].

Similarly, there is currently no implicated role of *TRPC6* in CSVD; however, some studies have shown that PSEN2 is known to interfere with the activity of TRPC6 without affecting agonist-induced Ca^2+^ release of other Ca^2+^ channels in HEK 293 T cells [47, 50]. As there were three paralogous *TRPC* genes identified either through TRAPD association or the candidate mutations approach, it shows a potential role of these genes in CSVD and neurodegenerative phenotypes. This may be related to the role they play in Ca^2+^ homeostasis in cells, something that is found to be dysregulated in AD-related pathologies. Furthermore, members of the TRPC family are found in rodent and human cerebrovascular tissue, cerebral arteries, and VSMCs, although clinical evidence linking these proteins to CSVD is limited [47, 51, 52].

Only *ERN1* was identified using the WES candidate mutation identification approach. The use of rare variant association testing using the TRAPD software was unsuccessful in identifying novel associations. In part, this is due to one of the limitations of this type of testing in this study as there were only 50 probands available for research. Future genetic investigations should involve segregation studies within families, functional studies using cell lines or animal studies to further confirm overlapping molecular processes in these diseases. Furthermore, increasing the population of the CADASIL-CSVD cohort should also be completed and other statistical burden tests should be repeated to utilise a more specific control dataset such as the UK Biobank or ASPREE dataset [53, 54]. This would allow for a better control population and may even aid in identifying other novel causes of disease [55, 56].

## Conclusion

In conclusion, analysis of the PANTHER Alzheimer’s disease – presenilin pathway genes in the CADASIL-CSVD cohort was successful in identifying novel candidate mutations that may be contributing to patient phenotypes. In particular, the mutations identified in *ERN1* across three separate unrelated individuals show a promising novel CSVD gene. There was also further evidence for potential links between AD and CSVD through the proportion of individuals shown as carriers for APOE-ε4. This work builds on theories of an overlapping genetic mechanism between Alzheimer’s disease and CSVD, however further studies need to be completed to truly start to elucidate an overlapping role between these two diseases.
